# The yield of tuberculosis contact investigation on relapsed TB patients and analysis of associated risk factors: Singapore’s experience

**DOI:** 10.1017/S0950268824000104

**Published:** 2024-01-17

**Authors:** Win M. Kyaw, Leo K.-Y. Lim, Jun Y. Tay, Jeffery L. Cutter, Deborah H. L. Ng

**Affiliations:** 1 National TB Registry; 2 National TB Programme, Singapore

**Keywords:** Tuberculosis, Contact investigation, Relapse case, TB Registry, TB infection

## Abstract

The yield of contact investigation on relapsed tuberculosis (TB) cases can guide strategies and resource allocation in the TB control programme. We conducted a retrospective cohort study to review the yield of contact investigation in relapsed TB cases and identify factors associated with TB infection (TBI) among close contacts of relapsed TB cases notified between 2018 and 2022 in Singapore. TB infection positivity was higher among contacts of relapsed cases which were culture-positive *for Mycobacterium tuberculosis* complex compared to those who were only polymerase chain reaction (PCR)-positive (14.8% vs. 12.3%). On multivariate analysis, after adjusting for age and gender of the index, gender, and existing comorbidities of contacts, factors independently associated with TBI were culture and smear positivity of the index (AOR 1.41, 95%CI 1.02–1.94), higher odds with every 10 years of increase in age compared to contacts below aged 30, contacts who were not Singapore residents (AOR 2.09, 95%CI 1.46–2.97), and household contacts (AOR 2.19, 95%CI 1.44–3.34). Although the yield of screening was higher for those who were culture-positive compared to only PCR-positive relapsed cases, contact tracing for only PCR-positive cases may still be important in a country with moderate TB incidence, should resources allow.

## Introduction

Tuberculosis (TB) remains a global public health threat with more than 10.5 million cases of TB disease globally with 1.6 million deaths [1]. Singapore is an island-city state with a resident population of 4.1 million which comprises citizens and permanent residents with a TB incidence of 30.7 per 100,000 population among residents in 2022 [2]. There was also a rise in incidence of newly diagnosed TB cases among non-residents in the past few decades [1]. Tuberculosis infection (TBI) is also not uncommon among resident population [3]. Singapore is located at the southern tip of the Malayan Peninsula in South-East Asia with a perpetual entry of foreigners from TB-endemic countries in Asia including 1.6 million long-staying non-residents who are permitted to work, study, or stay as dependents of non-residents in the country [4].

The National TB Control Programme was established in Singapore in 1958 with the setting up of the TB Control Unit and a National TB Registry. The programme was enhanced with the launch of the Singapore TB Elimination Programme (STEP) in 1997 and reorganized and renamed as National TB Programme (NTBP) in 2022. Being a notifiable disease under the Infectious Disease Act, clinicians are required to notify all cases of (clinical or laboratory diagnosed) TB identified to the National TB Registry which maintains a comprehensive electronic database of all notified TB cases nationwide. In addition, National TB Registry receives the laboratory reports including AFB (acid-fast bacilli) smears, AFB cultures, and molecular tests from the microbiology laboratories from both public and private hospitals daily nationwide.

Information collected include patient’s demographics, laboratory, relevant clinical information, treatment progress, and outcomes of all notified cases. The registry will then initiate contact tracing for cases that are bacteriologically confirmed. In the same database, the registry also maintains detailed contact investigation findings such as identified contacts with respective case details, screened contacts with screening results, and details of preventive therapy where relevant.

NTBP conducts contact tracing for TB cases using a concentric circle approach and extending further depending on the index’s infectivity and exposure. The period of contact exposure starts from 3 months prior to either the date of onset of symptoms (if any) or the notification date whichever is earlier. Contacts above 2 years of age are screened with interferon-gamma release assays (IGRAs), while those who are 2 years of age and below are screened with tuberculin skin test. Persons found to have TBI or have symptoms or signs of TB require further evaluation with chest X-ray. Those with abnormal chest X-rays are also required to undergo sputum evaluation.

Contact tracing is typically focused on new cases. However, contact tracing for relapsed cases who are smear- and culture-negative but PCR-positive, may not have contact tracing initiated as the PCR may be due to prolonged shedding, particularly if the first episode had occurred in recent years. Theron et al. had described in a retrospective retreatment population that false-positive GeneXpert results can persist up to 4 years [5]. Another study also described in a prospective population that GeneXpert can produce false-positive results in 8.72% of positive tests on respiratory samples for up to 18 months [6]. There are also challenges in initiating contact tracing again as contacts may have been screened recently and may not be keen to undergo screening again. Preventive treatment is also shown to have a protective effect for a duration of up to 10 years [7, 8]. Contact tracing for relapsed cases is therefore done on a case-by-case basis, with a focus on identifying vulnerable contacts or contacts who have not been screened prior.

Studies have been done globally to evaluate the yield of contact investigation done for newly diagnosed TB cases and the factors associated with increased yield [9–11]. A study done in a high-incidence country showed that retreatment index TB cases were 7.6 times higher yield of TB disease among their contacts than new cases [12]. However, there is very limited information on the yield of contact investigations based on the bacteriological status of relapsed cases in moderate TB incidence settings like Singapore. The yield of contact investigation on relapsed cases can guide strategies and resource allocation in building up the TB control programme. This study therefore aims to evaluate the yield of contact investigation in relapsed cases and identify factors associated with TBI among close contacts of relapsed TB cases in a moderate TB incidence country with a bacillus Calmette–Guérin (BCG)-vaccinated population.

## Methods

### Study design, population, and setting

We conducted a retrospective cohort study for the yield of TBI and TB disease among contacts of relapsed TB cases notified to the National TB Registry in Singapore between January 2018 and December 2022. A relapsed case was defined as a person previously treated for TB in Singapore or self-reported that treatment received before abroad, and it could be due a regrowth of the same strain that caused the previous TB episode or re-infection. IGRA conversion was defined as a change from negative result on initial IGRA test to a positive result on the succeeding test at 8 weeks from the last exposure date. General criteria for contact investigation for relapsed cases during the study period were those who had initial episode more than 10 years ago and bacteriologically confirmed (culture or PCR-positive) or clinically diagnosed pulmonary TB (PTB). For relapsed PTB cases who had only TB PCR-positive results, with an original episode less than 10 years ago, contact tracing was initiated on a case-by-case basis. Contact investigation was conducted for relapsed cases to identify potentially infected TB contacts during the infectious period. Identified contacts with prior TB treatment or chemoprophylaxis preventive therapy before screening were excluded from screening.

### Data collection

Epidemiological, clinical, and laboratory data of relapsed cases were extracted from the National TB Registry database. Data collected for relapsed cases included demographics (age, gender, nationality), type of TB, year of diagnosis, as well as baseline laboratory results (smear, culture, PCR). Data for all contacts identified and recorded in the registry were also extracted. Extracted contact data included demographics (age, gender, nationality), dates and results of screening tests, type of contact (close, casual), as well as exposure settings (household, workplace, school, healthcare). Close contacts are defined as contacts with cumulative exposure ≥8 h within the contact tracing period. In Singapore, TBI screening is done for close contacts of cases with *Mycobacterium tuberculosis* complex (MTC) culture- or PCR-positive and had cumulative exposure ≥8 h within the contact tracing period including congregate settings such as workplace, school, and healthcare institutions. Existing comorbidities recorded in the registry at the point of notification for relapsed cases and at the time of contact investigation for contacts, respectively, were also extracted. Contacts with TBI who eventually developed TB disease were identified by matching national registration numbers recorded in the registry.

### Statistical analysis

All data were analysed using Stata 16 (College Station, TX: StataCorp LLC). Pearson’s *χ* ^2^ test or the Fisher exact test for categorical variables was used to determine differences in the demographics, exiting comorbidities, and bacteriological results of relapsed cases and exposure details between contacts with TBI and those screened negative. Stepwise forward multiple logistic regression analysis was used to assess for factors associated with TBI. The odds ratios (OR) with 95% confidence intervals (CI) from regression analyses were determined, and P values less than. 05 were considered statistically significant. All statistically significant variables in univariate analysis were included in multivariate analysis together with other important variables to identify independent risk factors of TBI after accounting for multicollinearity. The best fit model was assessed using the receiver operating characteristics curve.

## Results

During the 5-year study period, 11,754 cases were notified to the National TB Registry. Of these, (6%) 702 were diagnosed as relapsed cases which consist of 77% (539) of residents and 23% (163) of long-staying non-residents in Singapore. Of these, 364 cases (52%) were bacteriologically confirmed. Contact investigations were done for 37% of relapsed TB cases (262 cases: 259, bacteriologically confirmed; 3, clinically/radiologically diagnosed). A total of 3,192 close contacts were identified during contact investigations, of which 67% (2129) agreed to screen for TBI. After excluding 3 indeterminate IGRA results with no repeat test, results from 2,126 contacts were left for further analysis. Among 2,126 contacts with a valid IGRA result, majority were residents (1,595, 75.1%), median age was 42 years (IQR, 30–58), and male and female ratio of 1:1. There were 13 contacts aged less than 5-year-old and identified as household contacts or frequent visitors to the household; however, none showed TBI or developed active TB disease during the study period. Distribution of exposure settings among all contacts is: household (556, 26.2%); healthcare/elderly care (491, 23.1%); workplace (439, 20.6%); frequent visitors to household (299, 14.1%); school (79, 3.7%); others (254, 12%); and information not available (8, 0.4%). Of 1,063 contacts who declined to screen for TBI, 581 were family members of relapsed cases, 481 were non-family members, and one contact of unknown relationship to the relapsed case.

### Characteristics of relapsed cases

The demographic profile of the relapsed cases is shown in Table 1. The median age (IQR) of the relapsed cases was 69 years (56–80), and median number of contacts (IQR) per index was 4 [2–8]. Majority were male (196, 74.8%), residents (223, 85.1%), and diagnosed with PTB (241, 92.0%). Of 262 relapse cases diagnosed during the 5-year study period, 71% (187 cases) had first TB episode more than 10 years ago. More than 88% (232 cases) of relapsed cases had first TB episode at least 5 years ago.

**Table 1.** Characteristics of the relapsed TB cases

Patient characteristics (*n* = 262)	*n* (%)
Age (years), median (IQR)	69 (56–80)
Median number of contacts per index (IQR)	4 (2–8)
Age group in years	
20–29	8 (3.1)
30–39	17 (6.5)
40–49	19 (7.3)
50–59	37 (14.1)
60–69	53 (20.2)
70–79	56 (21.4)
> = 80	72 (27.5)
Gender	
Male	196 (74.8)
Female	66 (25.2)
Residency status	
Resident	223 (85.1)
Non-resident	39 (14.9)
Year of diagnosis	
2018	54 (20.6)
2019	52 (19.8)
2020	48 (18.3)
2021	60 (22.9)
2022	48 (18.3)
Site of disease	
PTB	241 (92.0)
Both PTB and EPTB	20 (7.6)
EPTB	1 (0.4)
Time to relapse (years)	
0–2	15 (5.7)
3–5	24 (9.2)
6–10	36 (13.7)
>10	187 (71.4)

EPTB, extra-pulmonary TB; PTB, pulmonary TB.

### The yield of TBI screening

Among 2,126 contacts with a valid IGRA result, majority were residents (75.1%). Overall TBI positivity was 14.3% (304 contacts). TBI positivity among non-residents was higher (16.0%, 85 out of 530) than that of residents (13.7%, 219 out of 1,599). Based on the exposure settings, TBI positivity was highest among household contacts, 19.6% (109 out of 556), followed by healthcare/elderly care, 16.3% (80 out of 491); workplace, 11.4% (50 out of 439); and frequent visitors to household, 11.0% (33 out of 299), respectively. TBI positivity was the lowest among contacts exposed at school, 5.1% (4 out of 79).

The yield of contact investigations for relapsed cases is shown in Table 2. Of 1,865 contacts of 224 relapsed cases with culture-positive, TBI positivity among contacts was as follows: contacts of relapsed cases with culture-positive and PCR not done, 17.2% (10 out of 58 contacts); contact of relapsed cases with culture- and PCR-positive, 14.8% (223 out of 1,503 contacts); contacts of relapsed cases with culture-positive but PCR-negative, 14.1% (43 out of 304 contacts), respectively. TBI positivity among contacts of relapsed cases with only PCR-positive (culture-negative or not done) was 12.3% (23 out of 187 contacts). Although TBI positivity of 14.8% (276 out of 1865) was higher contacts of relapsed cases which were culture-positive (regardless of PCR result) compared to 12.3% (23 out of 187) of contacts of relapsed cases who were only PCR-positive, it was statistically not significant (*p* = 0.356). TBI positivity among 28 contacts of bacteriologically negative (culture-/PCR-negative) index was 11% (5 contacts).

**Table 2.** The yield of contact investigations for relapsed cases (bacteriologically confirmed or clinically/radiologically diagnosed)

Bacteriological results of index from respiratory specimens					TBI test results			
Smear	MTC Culture	PCR	No. of index	No. of Contacts screened	Positive	Negative	Indeterminate	TBI positivity (%)
±	+	ND	12	58	10	48		17.2
±	+	+	183	1,503	223	1,279	1	14.8
±	+	−	29	304	43	261		14.1
±	−	+	23	152	18	132	2	11.8
±	ND	+	7	35	5	30		14.3
−	−	−	3	46	5	41		10.9
+	−	−	4	28	0	28		0.0
+	ND	ND	1	3	0	3		0.0
Total			262	2,129	304	1822		

MTC, *Mycobacterium tuberculosis complex*; ND, not done; PCR, polymerase chain reaction; +, positive; −, negative.

### IGRA conversion and TBI cases who developed TB disease

General guideline adopted in our programme is to screen close contacts at baseline and at 8 weeks from the last exposure date (referred as window period). In our study, of 2,126 contacts with valid test results, 1830 were screened negative by first IGRA test. Of 1,279 contacts who need the second IGRA test, 1,196 contacts (93%) had a subsequent test. Of contacts with two IGRA tests, 33 contacts (31, relapsed culture-positive; 2, relapsed only PCR-positive) had IGRA conversion (2.8%). IGRA conversion was more common among contacts of relapsed cases who were only PCR-positive (3.0%, 2 out of 67 contacts) compared to contacts of relapsed cases with culture-positive (2.8%, 31 out of 1,114 contacts); however, it was statistically not significant (*p* = 0.922). Similarly, TB disease was more common among contacts of relapsed cases with culture-positive (0.8%, 14 out of 1864 contacts) compared to contacts of relapsed cases with only PCR-positive (0.5%, 1 out of 185 contacts) (*p* = 0.749).

Of 33 contacts with positive IGRA conversion, 29 (88%) completed preventive treatment. One contact who did not take preventive treatment developed TB disease. Of all screened contacts, 14 (0.7%) were eventually diagnosed with pulmonary TB within 3 months, and one contact progressed to TB disease within 15 months post-TBI diagnosis. Of 15 contacts who developed TB disease (contacts of 14 relapsed culture-positive index case; contact of 1 relapsed PCR-positive index case), four cases had bacteriological evidence to confirm the diagnosis (3, household contacts; 1, workplace contact). Nine contacts (60%) were household members of the relapsed cases, and 3 contacts (20%) were exposed at the workplace. Remaining 3 contacts were exposed at healthcare/elderly care or other setting and frequent visitor to the household, respectively.

### Factors associated with TBI diagnosis

Significant differences were observed in characteristics between the TBI and non-TBI groups (Table 3). On univariate analysis, contacts who were older, with existing comorbidity of diabetes, household contacts compared to non-household contacts were more likely to be diagnosed with TBI. However, frequent visitors to the household compared to non-frequent visitors to the household were less likely to be diagnosed with TBI. Multivariate analysis (Table 4) showed that contacts of both culture-positive and smear-positive relapsed cases had higher odds of being diagnosed with TBI (adjusted OR (AOR) 1.41, 95%CI 1.02–1.94) and time to relapsed was negatively associated with TBI diagnosis among contacts. Contact factors associated with TBI were non-residents (AOR 2.09, 95%CI 1.46–2.97), higher odds with every 10 years increases in age compared to contacts below 30-year-old, and household contacts of relapsed case (AOR 2.19, 95%CI 1.44–3.34) compared to non-household contacts after adjusting for age and gender of the index, gender, existing comorbidities of contacts, and exposure settings of contacts. The area under the ROC curve (AUC) was 0.69.

**Table 3.** Factors associated with LTBI diagnosis among close contacts: Univariate analysis

Variables	TBI (*n* = 304) (*n*, %)	Non-TBI (*n* = 1822) (*n*, %)	Odds ratio (95% CI)	*p*-value
Index (Relapse), Demographic				
Age (years), median (IQR)	67 (55–79)	67 (52–80)	1.00 (1.00–1.01)	0.427
Gender, Male	220 (72.4)	1,293 (71.0)	1.07 (0.82–1.41)	0.617
Interval (years) between first episode and relapse				
Year, median (IQR)	23 (10–37)	24 (12–41)	0.99 (0.98–0.99)	**0.047**
0–2 years	10 (3.3)	43 (2.4)	Ref	Ref
3–5 years	42 (13.8)	190 (10.4)	0.95 (0.44–2.04)	0.897
6–10 years	25 (8.2)	204 (11.2)	0.53 (0.24–1.18)	0.118
>10 years	227 (74.7)	1,385 (76.0)	0.70 (0.35–1.42)	0.329
Index (Relapse), Bacteriological result				
Culture and smear				
MTC Pos, Smear Neg	86 (28.3)	534 (29.3)	Ref	Ref
MTC Pos, Smear Pos	190 (62.5)	1,054 (57.9)	1.12 (0.85–1.47)	0.422
MTC Neg, Smear Neg	23 (7.6)	183 (10.0)	0.78 (0.48–1.27)	0.321
MTC Neg, Smear Pos	5 (1.6)	51 (2.8)	0.61 (0.24–1.57)	0.304
PCR and smear				
PCR Pos, Smear Neg	56 (18.4)	385 (21.1)	Ref	Ref
PCR Pos, Smear Pos	190 (62.5)	1,056 (58.0)	1.24 (0.90–1.70)	0.193
PCR Neg, Smear Neg	53 (17.4)	332 (18.2)	1.10 (0.73–1.64)	0.651
PCR Neg, Smear Pos	5 (1.6)	49 (2.7)	0.70 (0.27–1.84)	0.470
Contact, Demographic				
Age (years), median (IQR)	51 (34–66)	40 (29–56)	1.02 (1.01–1.03)	**<0.001**
Aged ≤30	52 (17.1)	504 (27.7)	Ref	Ref
Aged 31–40	53 (17.4)	414 (22.7)	1.24 (0.83–1.86)	0.295
Aged 41–50	44 (14.5)	256 (14.0)	1.67 (1.08–2.56)	**0.020**
Aged 51–60	43 (14.1)	312 (17.1)	1.33 (0.87–2.05)	0.185
Aged 61–70	62 (20.4)	176 (9.7)	3.41 (2.27–5.13)	**<0.001**
Aged ≥71	50 (16.5)	160 (8.8)	3.03 (1.98–4.64)	**<0.001**
Gender, Male	159 (52.3)	897 (49.2)	1.13 (0.89–1.44)	0.322
Non-resident	85 (28.0)	445 (24.4)	1.20 (0.91–1.58)	0.187
Contact, Co-morbidities				
Diabetes Mellitus	39 (12.8)	166 (9.1)	1.47 (1.01–2.13)	**0.043**
End Stage Renal Failure	13 (4.3)	61 (3.4)	1.29 (0.70–2.38)	0.415
Any malignancy	5 (1.6)	26 (1.4)	1.16 (0.44–3.03)	0.770
Contact, Exposure details				
Household	109 (35.9)	447 (24.6)	1.71 (1.32–2.21)	**<0.001**
Frequent Visitor to household	33 (10.9)	266 (14.6)	0.71 (0.48–1.04)	0.083
Healthcare/Elderly care	80 (26.3)	411 (22.7)	1.31 (0.96–1.79)	0.084
Workplace	50 (16.5)	389 (21.4)	0.72 (0.52–0.99)	**0.047**
School	4 (1.3)	75 (4.1)	0.31 (0.11–0.85)	**0.023**

MTC, *Mycobacterium tuberculosis complex*; PCR, polymerase chain reaction.

**Table 4.** Independent risk factors associated with LTBI among close contacts: Multivariate analysis

Variables	Adjusted OR (95% CI)	*p*-value
Index (Relapse)		
Age (years)	1.00 (0.99–1.01)	0.564
Male gender	1.02 (0.76–1.37)	0.903
Interval (years) between first episode and relapse	0.99 (0.98–0.99)	**0.045**
Index (Relapse), Bacteriological result		
MTC Pos, Smear Neg	Ref	Ref
MTC Pos, Smear Pos	1.41 (1.02–1.94)	**0.035**
MTC Neg, Smear Neg	0.79 (0.45–1.40)	0.421
MTC Neg, Smear Pos	0.84 (0.31–2.32)	0.758
Contact, Demographic		
Aged ≤30	Ref	Ref
Aged 31–40	1.17 (0.76–1.79)	0.471
Aged 41–50	1.92 (1.23–2.99)	**0.004**
Aged 51–60	1.81 (1.15–2.86)	**0.010**
Aged 61–70	4.86 (3.08–7.66)	**<0.001**
Aged ≥71	4.10 (2.51–6.69)	**<0.001**
Male gender	1.24 (0.95–1.61)	0.116
Non-resident	2.09 (1.46–2.97)	**<0.001**
Contact, Comorbidities		
Contact with Diabetes Mellitus	0.96 (0.62–1.47)	0.841
Contact with End Stage Renal Failure	1.33 (0.60–2.96)	0.481
Contact with any malignancy	0.90 (0.33–2.44)	0.835
Contact, Exposure details		
Exposure – Household	2.19 (1.44–3.34)	**<0.001**
Exposure – Frequent Visitor to household	1.23 (0.73–2.09)	0.433
Exposure – Healthcare/Elderly care	1.47 (0.90–2.39)	0.122
Exposure – Workplace	0.98 (0.60–1.60)	0.937
Exposure – School	0.51 (0.17–1.57)	0.242

MTC, *Mycobacterium tuberculosis complex*; PCR, polymerase chain reaction.

Bold values to highlight the statistically significance of p-value.

## Discussion

This is the first retrospective cohort study in Singapore to evaluate the yield of contact investigation based on the bacteriological status of relapsed TB cases. During the 5-year study period, TBI yield was 14.3% and TB disease yield was 0.7%, respectively, among close contacts of relapsed TB cases. This finding of TBI positivity is high in a country with TB incidence rate of 30.7 cases per 100,000 population when compared to high-income setting in South-East Asia where a TB incidence of 57 per 100,000 population with TBI positivity of 9.9% [9]. This finding in our study was largely contributed by non-residents as TBI positivity among non-residents was higher than that of residents (16.0% vs. 13.7%) among all screened contacts although it did not reach the statistical significance. Notably, the proportion of residents was higher than that of non-residents among contacts who developed TB (72% vs. 28%).

Our study shows respective TBI yield in different exposure settings. Household contacts who had shared accommodation with the relapsed had higher yield of more than 20% compared to non-household contacts. This result concurs with previous finding of household contacts being at increased risk of TBI diagnosis [9]. Studies have also reported that tuberculosis disease was common among household contacts of infectious index cases [9, 13, 14]. All household contacts of relapsed pulmonary TB cases should be screened for TBI.

In our study, the proportion of contacts diagnosed with TBI was considerably higher among contacts of culture-positive relapsed patients than among contacts of only PCR-positive patients. Index with both culture-positive and smear-positive was also identified as a risk factor for TBI among contacts in our study. Previous studies have also shown that smear positivity of the index case was a risk factor for TBI [15, 16]. Our study also shows IGRA conversion and TB disease were more common among those who were culture-positive compared to those who were only PCR-positive, although they did not reach statistical significance. However, this affirms the fact that relapsed cases with culture-positive are more infectious than those who are only PCR-positive. In addition, we found that TBI positivity among contacts of smear-negative relapsed cases was 11%. This finding highlights a potential need to further understand the infectivity of smear-negative relapsed cases and to consider potential implications on guidelines for contact investigation.

In this study, we uncovered the factors associated with TBI yield among relapsed TB cases in a country with moderate TB incidence. We found that the odds of TBI yield increased with the age of contacts, and household contacts had higher yield of TBI. Demographic risk factors among contacts were comparable to that of relapsed cases, which was anticipated. Age is a well-known risk factor for TBI [17], and also a risk factor associated with TBI among household contacts [18]. Studies have shown that high TBI among household contacts with more 50% of household contacts have TBI [18], and 13% of household contacts were diagnosed with TB disease during the 3-year follow-up [19].

We also found that the interval between first episode and relapsed of index was negatively associated with TBI among contacts. Contact tracing initiation for relapsed cases with both culture-positive and smear-positive results should be considered regardless of time to relapsed from first TB infection episode. Notably, non-residents were identified as a risk factor associated with TBI. These non-residents might have originated from the high TB-incidence neighbouring countries in South-East Asia, and they might already have TBI before they were identified as close contacts. In Singapore, foreign workers are screened for TB disease on their arrival or pre-entry but not screened for TB infection. TB infection screening strategy should be implemented for specific demographic groups plausibly on new migrants arriving from high TB incidence countries.

Our study shows 67% of identified contacts agreed to screen for TBI. Of contacts who declined contact screening, more than 50% were family members of relapsed cases. More efforts are required to minimize refusal for contact screening such as mandatory contact screening for household family members as well as to conduct further studies to understand the knowledge and perception on TB infection and TB disease among identified contacts especially those declined the screening. Contact investigations should be done to ensure early detection of TBI and initiation of preventive treatment to reduce the risk of development of TB disease as well as the risk of disease transmission in a country.

This study had a few strengths. First, data were collected electronically by the staff who interviewed the contacts and oversaw the contact investigation for the case on routine basis. This lessened the data transfer error, and structured data with predefined options help in retrieving analysable data. Second, our study uses the data from the National Registry database which is a centralized population-based containing data for both index cases and identified contacts upon contact investigation.

Last, but not least, findings from our study contribute to the literature on the yield of contact investigations based on the bacteriological status of relapsed cases and are also likely to be generalizable to similar TB settings.

Our study also had some limitations. Contact investigation was done for only 37% of all notified relapsed cases based on the contact tracing criteria during the study period. This might impact on findings of the TBI yield and might lead to under estimation of the TBI rates among contacts of relapsed cases. However, contact investigation was done for more than 70% (259 out of 364) of bacteriologically confirmed relapsed TB cases notified during the study period which might have lessened the impact on findings. Among identified close contacts, 33% did not undertake the screening for TBI. Because of the retrospective nature of the study, we were unable to collect the missing information. This made it difficult to evaluate the differences in demographic characteristics of identified contacts between those who complete the screening and who did not.

## Conclusion

The TB yield among contacts of relapsed TB cases was higher for relapsed TB cases who were culture-positive compared to only PCR-positive cases. Nonetheless, contact investigation for only PCR-positive cases may still be important in a country with moderate TB incidence. Household contacts should all be screened regardless of relapsed cases with culture- or PCR-positive. Further studies are recommended to understand the knowledge and perception on TB infection and TB disease among identified contacts especially those declined the screening. Strategy to invest in TBI screening of immigrants from high TB incidence countries ought to be implemented, should resources allow.

## Data Availability

Due to the ethical reason, data sharing is not applicable.
